# A novel group of genes that cause endocrine resistance in breast cancer identified by dynamic gene expression analysis

**DOI:** 10.18632/oncotarget.28225

**Published:** 2022-04-06

**Authors:** Arvand Asghari, Katherine Wall, Michael Gill, Natascha Del Vecchio, Farnaz Allahbakhsh, Jacky Wu, Nan Deng, W. Jim Zheng, Hulin Wu, Michihisa Umetani, Vahed Maroufy

**Affiliations:** ^1^Center for Nuclear Receptors and Cell Signaling, Department of Biology and Biochemistry, University of Houston, Houston, TX 77204, USA; ^2^Department of Biostatistics and Data Science, School of Public Health, UTHealth, Houston, TX 77030, USA; ^3^Chicago Center for HIV Elimination, University of Chicago, Chicago, IL 60637, USA; ^4^Clinical Cancer Prevention Department, University of Texas MD Anderson Cancer Center, Houston, TX 77030, USA; ^5^School of Biomedical Informatics, UTHealth, Houston, TX 77030, USA; ^6^Health Research Institute, University of Houston, Houston, TX 77204, USA; ^*^These authors contributed equally to this work

**Keywords:** breast cancer, triple negative breast cancer, gene expression profiling, endocrine resistance, gene clustering

## Abstract

Breast cancer (BC) is the most common type of cancer diagnosed in women. Among female cancer deaths, BC is the second leading cause of death worldwide. For estrogen receptor-positive (ER-positive) breast cancers, endocrine therapy is an effective therapeutic approach. However, in many cases, an ER-positive tumor becomes unresponsive to endocrine therapy, and tumor regrowth occurs after treatment. While some genetic mutations contribute to resistance in some patients, the underlying causes of resistance to endocrine therapy are mostly undetermined. In this study, we utilized a recently developed statistical approach to investigate the dynamic behavior of gene expression during the development of endocrine resistance and identified a novel group of genes whose time course expression significantly change during cell modelling of endocrine resistant BC development. Expression of a subset of these genes was also differentially expressed in microarray analysis of endocrine-resistant and endocrine-sensitive tumor samples. Surprisingly, a subset of those genes was also differentially genes expressed in triple-negative breast cancer (TNBC) as compared with ER-positive BC. The findings suggest shared genetic mechanisms may underlie the development of endocrine resistant BC and TNBC. Our findings identify 34 novel genes for further study as potential therapeutic targets for treatment of endocrine-resistant BC and TNBC.

## INTRODUCTION

Breast cancer (BC) is the most prevalent type of cancer in women. More than 280 000 new US cases of breast cancer and 43 000 deaths were projected for 2021 [1]. While current five-year survival rates of BC have reached 90%, it remains the second leading cause of cancer-related deaths in women overall [1]. Treatment options for breast cancers typically involve surgical resection of the tumor followed by drug treatment based on the cancer type [2]. Breast cancers are categorized into subtypes based on hormone receptors (HR), namely estrogen receptor (ER) and progesterone receptor (PR), and human epidermal growth factor receptor 2 (HER2) expression status. Nearly 70% of breast cancers express the estrogen receptor (ER^+^) without overexpression of HER2 (ER^+^/HER2-negative BC, luminal A breast cancer) [2]. While 13% of breast cancers are HR^+^/HER2^+^ (luminal B), more than 13% are HR^−^/HER2^−^, also referred to as triple-negative breast cancer (TNBC). TNBC accounts for around 15% of breast cancer cases, characterized as one of the most aggressive types of breast cancer with no established therapy options yet [2]. While five-year survival rates of luminal A breast cancer are around 90%, the five-year survival rate of TNBC is more variable and, on average, lower at 77%, with patient prognosis typically poor [2].

ER exists in two isoforms, ERα (*ESR1*) and ERβ (*ESR2*), with ERα as the dominant form in BC. ER acts as a transcription factor mediating gene expression and as a signaling molecule, inducing kinase pathways and regulating cell growth in cultured breast cancer cells [3]. Given that 80% of breast cancers express ER, ER-targeted endocrine therapies are a core component of systemic therapy. Endocrine therapies include selective ER modulators (SERMs) such as tamoxifen, selective ER down-regulators (SERDS) such as fulvestrant, and aromatase inhibitors (AIs) targeting estrogen biosynthesis. Endocrine therapies have been successful at improving cancer outcomes; however, the development of endocrine resistance, or resistance to inhibition of ER actions, remains a roadblock in breast cancer treatment. Many patients have intrinsic resistance to endocrine therapies. Only 30% of patients with metastatic disease see initial regression with endocrine therapies [4], however, in almost all patients the resistance develops eventually, and tumors frequently recur. Moreover, more than 20% of patients who present with early breast cancer will develop endocrine resistance throughout treatment [4, 5].

Resistance to endocrine therapies has been traced to mutations in *ESR1*, alterations in receptor tyrosine kinases such as HER2, and alterations in signaling pathways such as the MAPK pathway. Point mutations in the ligand-binding domain (LBD) of *ESR1*, have been shown to cause endocrine resistance [5–8]. The most common mutations, Y537 and D538, lead to constitutive ligand-independent activation of ERα [8, 9]. These mutants are less sensitive to fulvestrant or tamoxifen [7]. LBD mutations in *ESR1* have been found in around 20% of metastatic ER^+^ cancers after endocrine therapy [7, 10]. *ESR1* mutations in circulating tumor DNA (ctDNA) have been found in 36% of patients with metastatic tumors following AI treatment [11]. Gene fusions of the *ESR1* DNA binding domain to the C-terminus of other proteins, though rare, can promote ligand-independent ERα activity [12].

*HER2* mutations occur in 2.4% of primary tumors and 6.7% of metastatic tumors [9]. Activating mutations of *HER2* can confer resistance to estrogen deprivation and fulvestrant treatment; fulvestrant sensitivity can be restored with HER2 inhibitors [13]. HER2 amplification is less common, occurring in 0.8% of primary and 2.1% of metastatic tumors [9], Amplification of HER2 has been linked to tamoxifen resistance, through the hyperactivation of MAPK [14]. Currently, ER^+^/HER2^+^ breast cancers are treated with both antiestrogens and HER2 inhibitors [9]. However, for patients with endocrine-resistant breast cancers, treatments are limited as the development of endocrine resistance is not well understood. Existing scientific literature focuses on the role of *ESR1*, receptor tyrosine kinases, and their signaling pathways; however, genetic mutation of these genes comprises a small percentage of cases. In at least 60% of cases, additional factors must play important roles in the resistance development process as those cases show intact *ESR1* and no up- or down-regulation in other signaling pathways [10].

In this study, we explored the dynamic behavior of the entire gene population to identify novel genes that play fundamental roles in the development and progression of endocrine-resistant breast cancer. We used the (i) time course gene expression patterns cells that develop endocrine resistance over time [15], (ii) expression of patient data from endocrine-resistant tumors compared to endocrine-sensitive ones, and (iii) expression data for TNBC compared to luminal A breast cancer to determine whether similarities among different BC subtypes were present and, if so, to identify key candidate genes associated with underlying mechanisms.

## RESULTS

### Selection of cell-based model for endocrine resistance: LTED MCF7 cells display similarity to patient tumor data

First, we sought to identify the optimal cell-based model for development of endocrine resistance. For this purpose, we used three datasets from the GEO repository, summarized in Table 1. First, we compared the gene expression patterns of several cell models before and after establishment of resistance, utilizing datasets GSE20361 and GSE111151, to those of endocrine-resistant and -sensitive tumors in the GSE87411 patient tumor dataset [16]. To the best of our knowledge, it represents the only publicly available expression dataset for endocrine-resistant tumors. It includes measurements from baseline and follow-up after 2–4 weeks exposure to aromatase inhibitors for 109 subjects [16]. Evaluating patient response to treatment, tumors were biopsied, analyzed, and categorized into endocrine-resistant and endocrine-sensitive categories. This data was compared to potential cell models.

**Table 1:** Summary of incorporated datasets from the GEO repository

GEO Dataset	Cell line(s)	Experimental exposure	Observed time points per sample
GSE20361	MCF7	Long-term estrogen deprivation	0 3 15 30 90 120 150 180 days
GSE87411	MCF7 ME16C	Neoadjuvant aromatase inhibitor treatment	0 days (pre-treatment) 14–28 days (post-treatment)
GSE111151	MCF7 T47D ZR75-1 BT474	Control, Tamoxifen exposure (8–12 months)	0 days 8–12 months (post-exposure)

**Figure 1:**
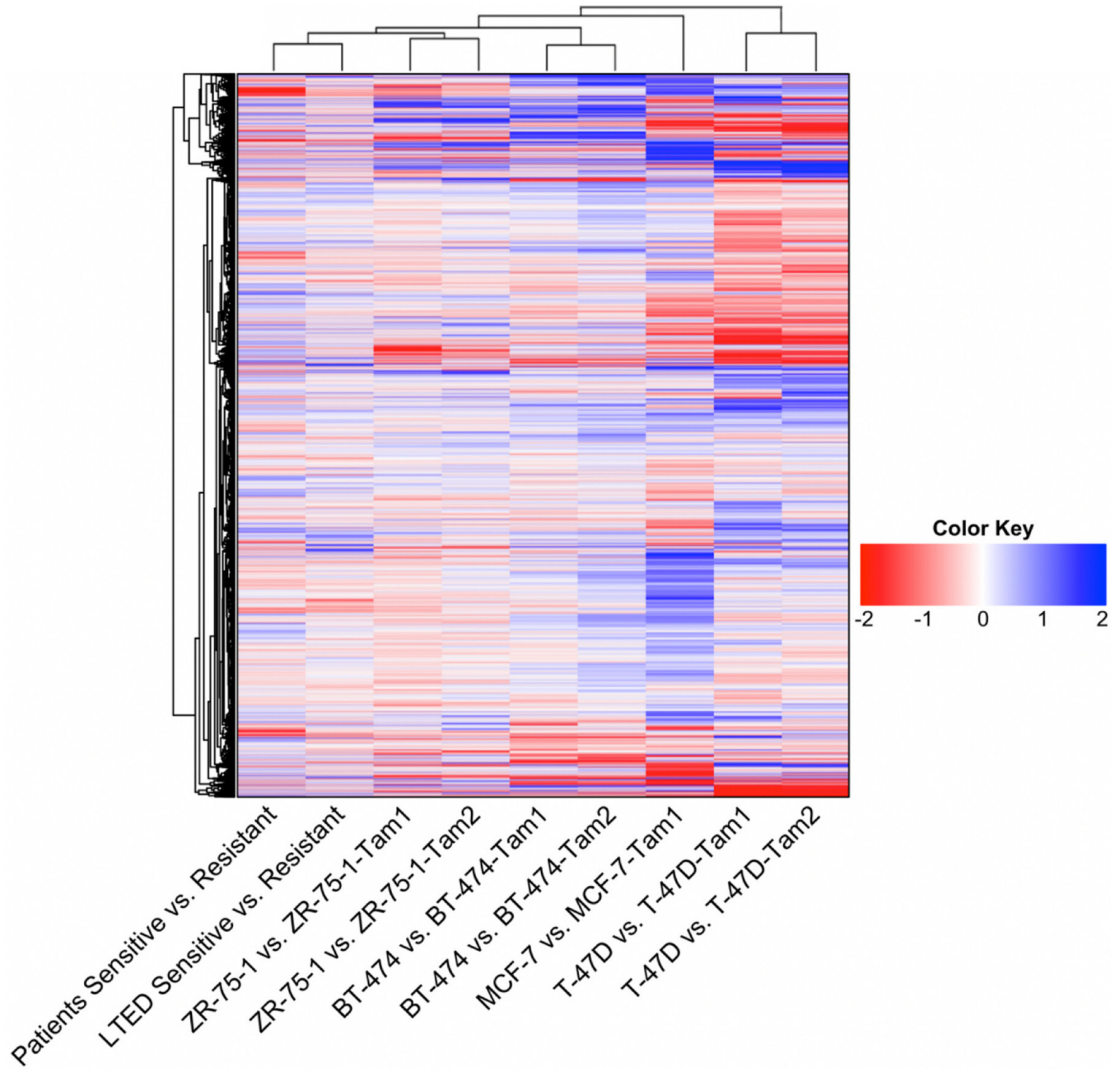
Heatmap of the global gene expression patterns between cell-based models of endocrine resistance and patient data. Data for the LTED cell model was obtained from GSE20361. Data for patient’s responses to hormonal therapies was obtained from GSE87411. The rest of the cell model data was obtained from GSE111151. For cell models ZR75-1, BT474, and T47D the data consisted of two populations of tamoxifen-resistant cell lines, each represented above. The dendrogram plots similarity measures between datasets, showing that the LTED cell model was most similar to patient data.

Possible models were inspired by GSE20361 and GSE111151. One possible model came from the GSE20361 dataset, which was obtained through culturing MCF7 cells in estrogen-deprived medium, called long-term estrogen-deprived (LTED) cells, which developed resistance to endocrine therapy [15]. In contrast, GSE111151 contained multiple models of tamoxifen resistance in several breast cancer cell lines: MCF7, T-47D, ZR-75-1, and BT-474. Their expression data was observed at two time points, before and after resistance development [17]. We computed the distance, or similarity, of each to the patient data, visualized as a heatmap in Figure 1. By applying hierarchical clustering to these possible models, we found the gene expression patterns of LTED cells were most similar to the patient data, visually shown as the shortest distance, closest cluster, in the heatmap’s overhead dendrogram. ZR-75-1 tamoxifen-resistant cell lines were next closest, followed by BT-474, MCF7, and T-47D tamoxifen-resistant cell lines. Therefore, we decided to use LTED cells as the most suitable cell line model for studying endocrine resistance in breast cancer.

### Estrogen-deprived MCF7 cells show four dominant expression patterns with unique cell functions

To better understand the process of acquiring endocrine resistance and its underlying gene expression patterns, we re-used the time course GSE20361 dataset for bioinformatic analysis. This data contained RNA measurements from the deprived MCF7 cells after 0, 3, 15, 30, 90, 120, 150, and 180 days. We applied our recently developed statistical pipeline to the dataset to find dynamically regulated genes active in the process of endocrine resistance development and progression [18]. The pipeline provides three main functions. First, statistical hypothesis testing determines a set of dynamic response genes (DRGs) that exhibit signiﬁcant changes over time. Next, these DRGs are clustered into gene response modules (GRMs), sets of DRGs with similar time course expression patterns. Finally, the GRMs associations and regulatory effect are analyzed as a gene regulatory network using ordinary differential equations.

**Figure 2:**
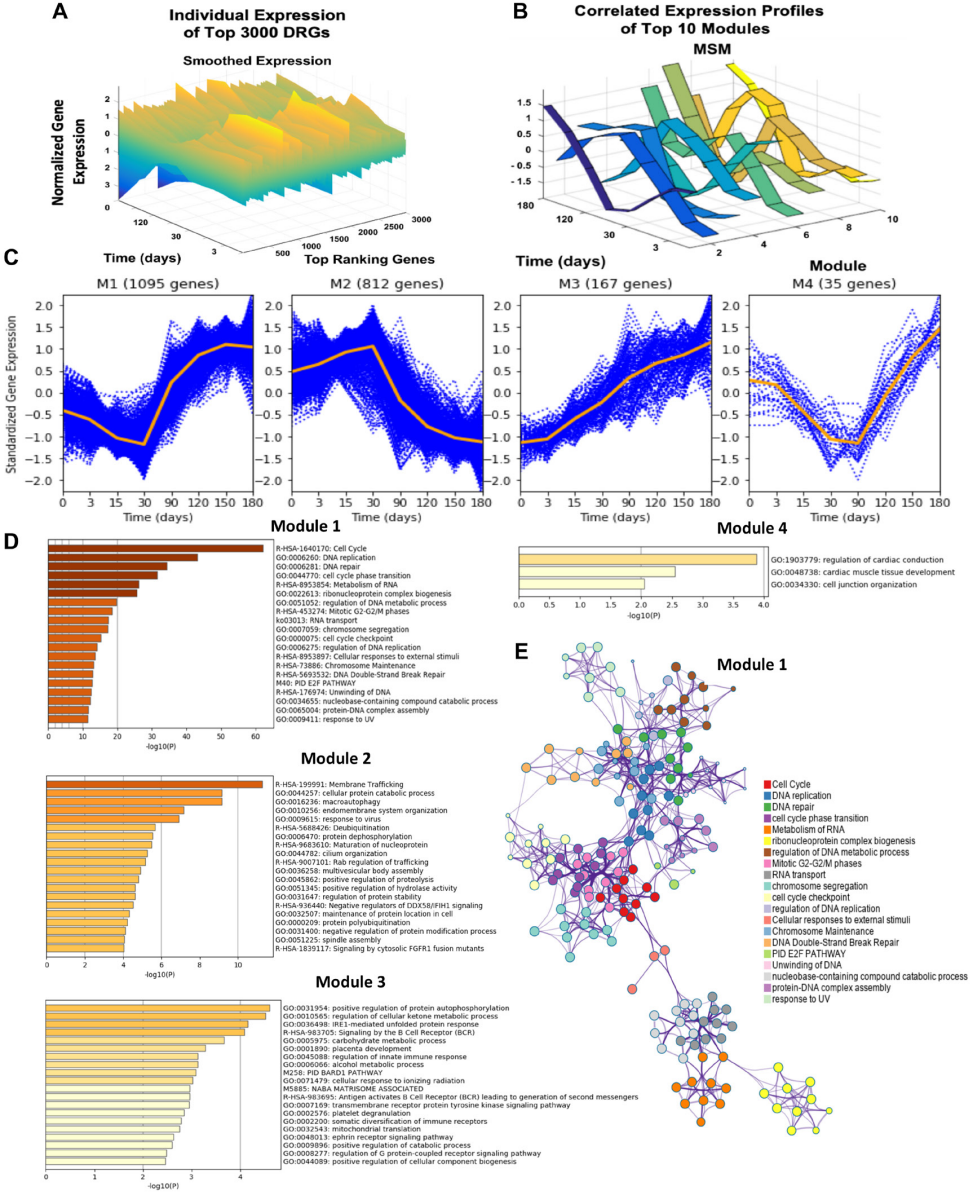
Dynamic gene expression analysis results, gene expression trends of top four modules, and network analysis of the top four modules DRGs using Metascape. (**A**) Presents the individual time-resolved expression trends for the top 3 000 dynamic response gene probes. These 3 000 probes are comprised of 2 305 identifiable unique genes. Y-axis represents the normalized expression of genes as described in the methods section. (**B**) Illustrates the multiple-sampling method (MSM) results of mean expression trends for the 10 largest modules. (**C**) The time-resolved expression trends for the top four largest modules are shown. Each blue dotted curve represents one gene, and the middle orange line illustrates the mean expression at each time point. (**D**) Presents the significantly enriched terms in modules 1–4 based on the corresponding Fisher’s Exact Test *p*-values. (**E**) Presents the statistically enriched terms in module 1 hierarchically clustered into a tree based on Kappa-statistical similarities among gene memberships.

Starting with 54 675 probes in the GSE20361 microarray data, the pipeline detected 14 693 probes as DRGs. The 14 693 DRGs were ranked based on F-statistic, and the top 3 000 DRGs were selected for correlation-based iterative hierarchical clustering [18, 19]. The top 3 000 DRGs were clustered into 20 distinct GRMs. Each GRM is a cluster of genes exhibiting a high degree of expression profile trajectory correlation. Module sizes ranged from containing 1 gene to 1 095 correlated genes. The trajectories for the 10 largest modules show evident differences from one module to another, which emphasizes the resolution of our clustering method (Figure 2A, 2B).

Though 20 modules were identified, the largest four modules dominate, accounting for 92% of the top DRGs (Figure 2C). The largest, module 1, contained 1 095 unique genes whose expression was characterized by a gradual downregulation in expression from day 0 to 30, at which point the pattern shifted, revealing sharp upregulation from day 30 to 150. Module 2, comprised of 812 genes, showed nearly opposite behavior, demonstrating gradual upregulation until day 30, after which sharp downregulation was observed. By gene enrichment analysis against pathway and ontology annotations, module 1 was significantly enriched for cell division, DNA replication, DNA-dependent DNA replication, and cell cycle functions. The interaction between these different gene ontologies is also visualized in a tree cluster in Figure 2E. Module 2 was significantly enriched in membrane trafficking, cellular protein catabolic processes, and macro-autophagy. Module 3 was enriched in positive regulation of protein autophosphorylation and regulation of cellular ketone metabolic processes while module 4 was associated with cell junction organization (Figure 2D, 2E). After identifying genes displaying significant changed during the development of endocrine resistance, clusters of genes with correlated time-resolved profile trajectories were found to be significantly enriched in unique cellular functions.

### Dynamic gene expression analysis of LTED data overlaps with endocrine-resistant patient data and with genes regulated in TNBC

We further compared the top DRGs to two additional datasets to identify candidate genes with highly active roles in endocrine resistance.

**Figure 3:**
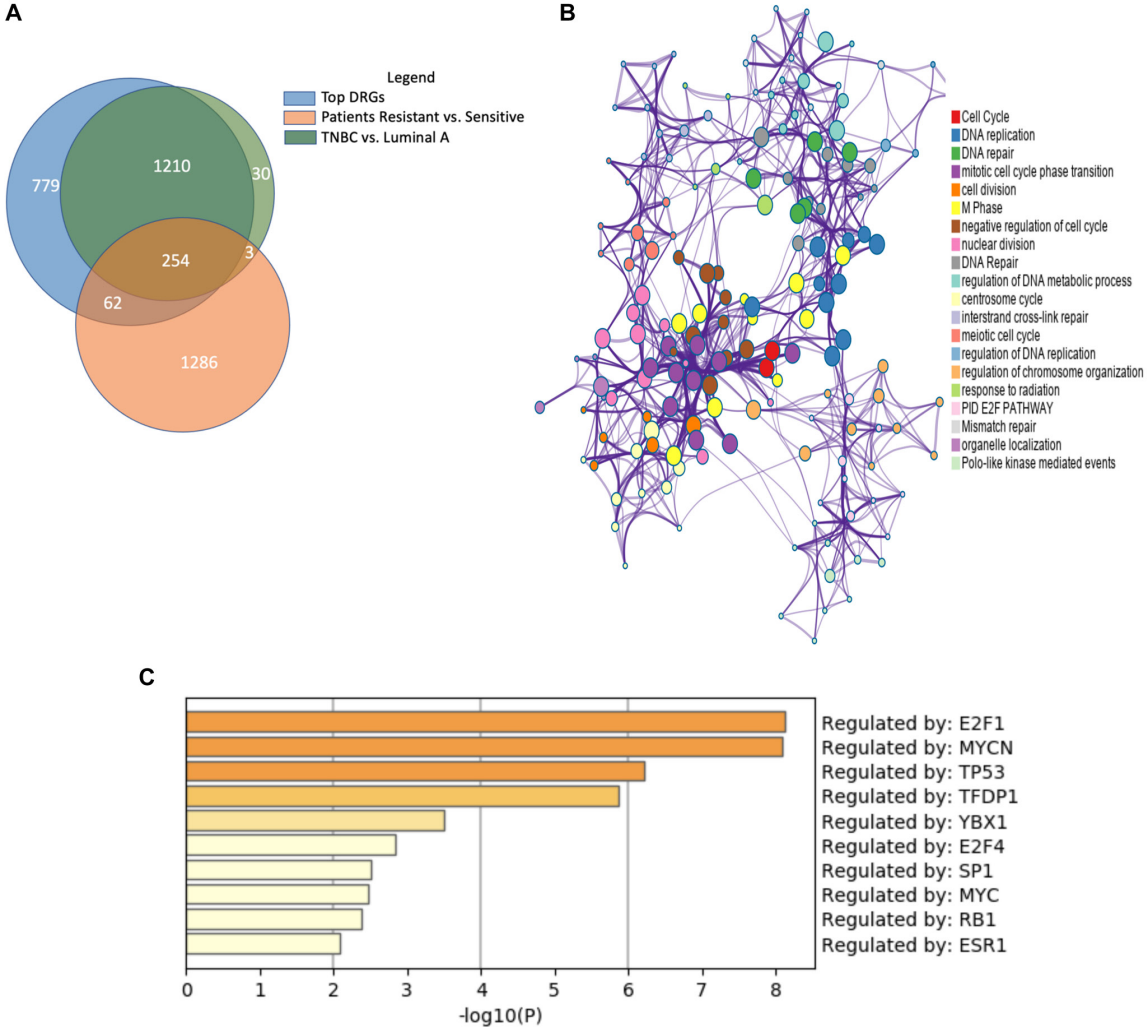
Similar gene expression patterns between endocrine-resistant breast cancer and triple-negative breast cancer. (**A**) Venn diagram depicts the shared significantly regulated genes between endocrine-resistant and TNBC and patient data. (**B**) Enrichment analysis of ontologies and pathways for the shared genes. (**C**) Transcription factor (TF) analysis of the common genes shows the majority of genes are regulated by E2F1, MYCN, and TP53 TFs.

The first dataset we filtered against was GSE87411, the patient data used for cell model selection. To find significant differentially expressed genes involved in regulation of endocrine-resistant breast cancer tumors versus -sensitive tumors, we compiled, analyzed, and compared results [20]. Among the differentially expressed genes, 984 genes were significantly upregulated in endocrine-resistant tumors while 621 genes were significantly downregulated. We then compared against the DRGs found in dynamic gene expression analysis, selecting 318 common genes for further analysis (Figure 3A).

Next, we incorporated a third dataset. Like endocrine-resistant breast cancer patients, the tumor cells of TNBC lack of hormone receptors, rendering them resistant to conventional endocrine therapies. To examine the potential for shared expression patterns, we analyzed UALCAN data from the Cancer Genome Atlas project. Between luminal A breast cancer and TNBC, we identified 1 497 genes significantly, differentially expressed. Surprisingly, 97.8% of genes significantly expressed in the TNBC vs. Luminal A gene set were present among the DRGs. More than 80% of the genes common to the DRGs and endocrine-resistant patient data were also significant in TNBC vs. Luminal A.

The common gene set between the three analyses was comprised of 254 genes. We posit these genes are important to the development of endocrine resistance, estrogen deprivation, and TNBC (Figure 3A). Enrichment analysis of the shared 254 genes showed significant enrichment for cell cycle, cell division, and DNA repair pathways, all signatures affected in cancer cells (Figure 3B). Enriched pathways related to DNA repair support the importance of the DNA repair system to account for mutations in TNBC and endocrine resistant breast cancer [21]. One of the common genes was ribonuclease H2 subunit A (*RNASEH2A*), a gene known for upregulation in cancers. RNASEH2A is a mediator of the removal of lagging-strand Okazaki fragment RNA primers, thus it can be integral in the proliferation of both triple-negative and endocrine-resistant breast cancers [22]. Moreover, many of the common genes are regulated by transcription factors E2F1, MYCN, and TFDP1, all important transcription factors in TNBC (Figure 3C). This finding provides evidence supporting the hypothesis that TNBC tumors share similarities with endocrine-resistant breast cancer.

### Top candidate genes responsible for endocrine-resistant development include MCM family, RAD51, CAV1, and CCNE1

**Figure 4:**
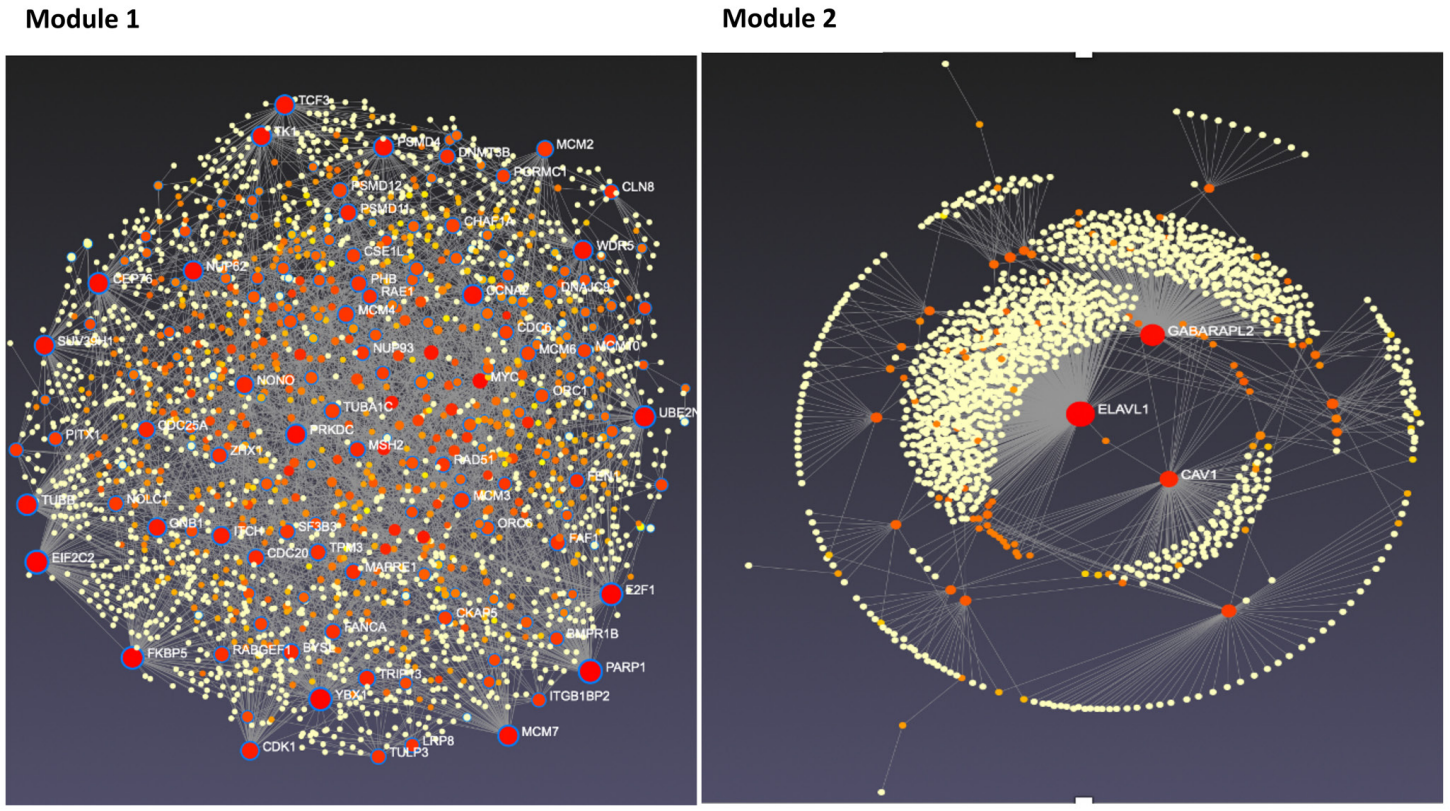
Tissue-specific protein-protein interaction network for modules 1 and 2 candidate genes. Candidate genes from modules 1 and 2 were analyzed using NetworkAnalyst. Resulting interaction networks are shown below. Red and orange circles represent the candidate proteins while yellow represent proteins that interact with our candidate proteins. The size of the circle represents the number of interactions. The scale of the module 1 and 2 varies due to module size.

To further refine the genes common to endocrine resistance development and progression, we utilized several bioinformatic approaches designated to rank and prioritize the 254 common genes. Using generated gene-gene and protein-protein networks, we identified genes with the highest number of network neighbors as master regulators. These master regulators among the common 254 genes formed our main candidates for their potential in holding essential roles in the development of endocrine resistance in LTED-MCF7 cells. Figure 4 features the networks for module 1 and module 2 [23–26]. Thereby, we were able to narrow the list of candidates to 34 genes presented in Supplementary Table 1.

**Figure 5:**
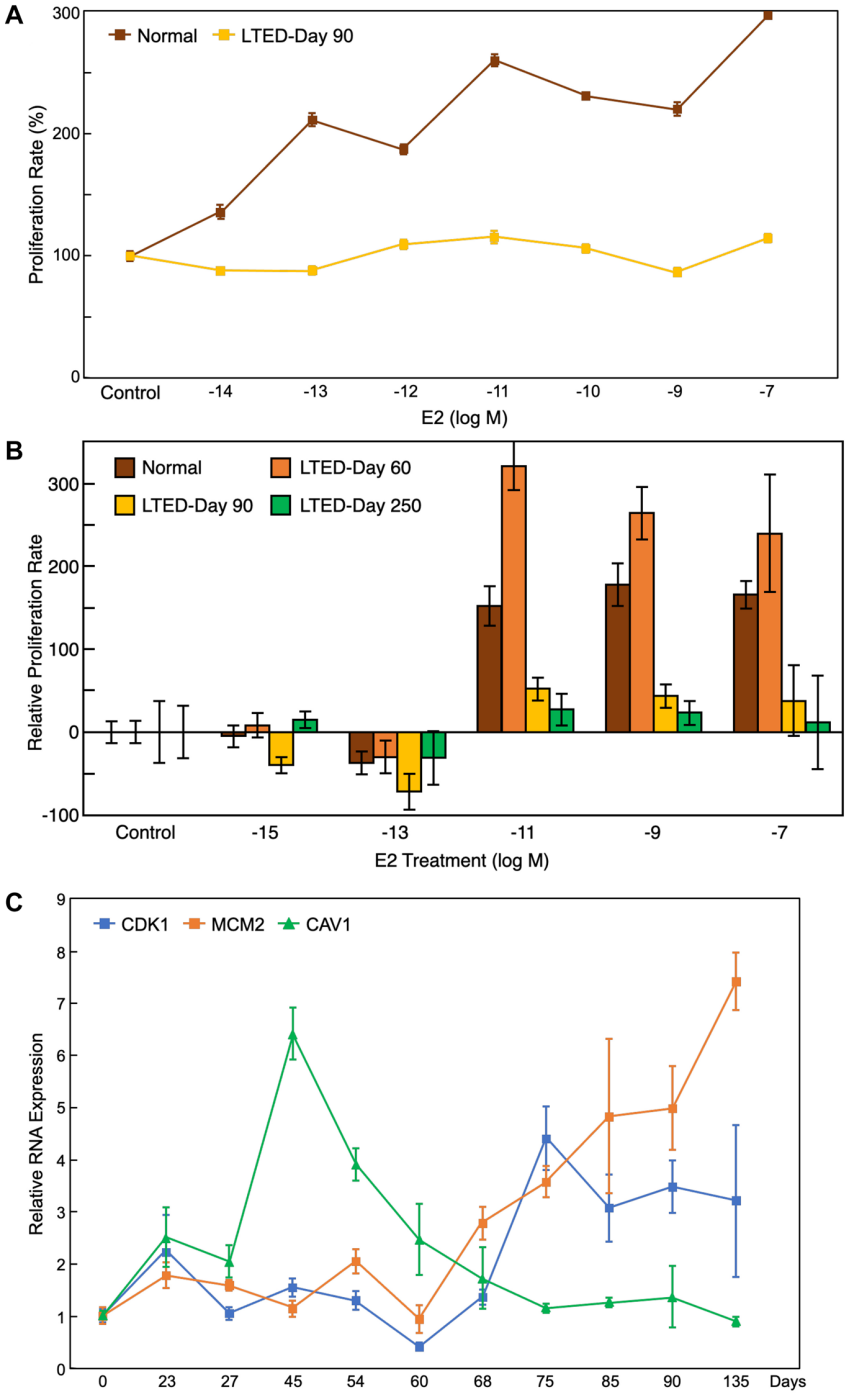
Proliferation assay and qRT-PCR results support bioinformatic findings. (**A**) Proliferation assay of the LTED cells at day 90 and normal MCF7 cells under different treatment doses of E_2_ (from 10^–14^ to 10^–7^ M) showed that LTED cells were not responsive to estrogen treatment. (**B**) Proliferation assay of the LTED cells at different days under several doses of E_2_ (from 10^–15^ to 10^–7^ M) show no responsiveness to E_2_ treatment by day 250. (**C**) qRT-PCR results for MCM2, CDK1, and CAV1 confirmed that the gene expression patterns *in vitro* match their expected gene expression patterns from microarray bioinformatics analysis. All values represent mean ± SEM (*n* = 3–6).

Master regulators were found across the top four modules but were predominantly represented by modules 1 and 2. Among the genes in module 1, we found *PARP1* and *E2F1*, recently discovered important genes for endocrine resistance in breast cancer, supporting the validity of our approach. We also found minichromosome maintenance (MCM) family genes as important genes, namely *MCM2* and *MCM7*, *RAD51*, and TCF3 (Figure 4), which yet to be thoroughly studied [27–29]. MCM family genes (*MCM2-7*) form an MCM complex protein that functions as a DNA replication licensing factor and plays a central role in eukaryotic DNA replication [30]. Recent evidence suggests that blocking the expression of these genes can lead to the inhibition of the growth of tamoxifen-resistant cancer cells. This evidence perfectly matches the expression pattern of these genes in our dataset, as these genes are significantly upregulated during the endocrine resistance process in breast cancer cells [31]. RAD51 overexpression, a key protein of homologous recombination, is also linked to overall poor survival and endocrine resistance in breast cancer, although the exact underlying signaling pathways are not well understood yet [32]. As for TCF3, while there is no study on its role in endocrine resistance, it is important for breast cancer differentiation, development, and prognosis [33]. Genes from in module 2 were slightly upregulated by day 30 and significantly downregulated afterward (Figure 5). In line with our data, reports show downregulation of CAV1 as an important step in breast cancer development and resistance to endocrine therapies [34]. Further studies are warranted. Additional important candidate genes were selected from modules 3 and 4, including *ATG3*, *CCNE1*, and *MFAP4.*

### Validation of LTED MCF7 profiles shows cells gain resistance at day 90

As validation of the bioinformatic findings, we established the LTED MCF7 cell model by culturing human breast cancer MCF7 cells in estrogen-depleted growth media for an extended period. This cell model recapitulated acquired resistance to aromatase inhibitors in postmenopausal women. Using the same experimental settings as GSE20361, we conducted gene expression analysis and extended the sample collection period [15]. After the initial quiescence state of around 30 days when the cell’s growth rate was minimal, cells started to adapt with estrogen depletion and regain their growth ability while losing their responsiveness to 17β-estradiol (E_2_). At around day 90, LTED cells showed complete loss of response to E_2_, characterized by a loss of ability to respond to estrogen stimulation to increase growth rate, while basal growth rate reached similar levels as those of parental MCF7 cells cultured in estrogen-rich media (Figure 5A). We maintained the LTED cells for another 6 months. After a period of super-sensitivity to estrogen at low doses (10^−11^ to 10^−13^ M), which happened around day 150, cells became completely unresponsive to estrogen treatments at all concentrations by day 250 (Figure 5B). These results are consistent with the previous reports and follow a similar timeline [15, 35–38].

To validate the gene expression profiles of the 34 candidate genes, we collected samples of LTED cells at different time points (from day 0 to day 135) and measured the expression of some of the candidate genes using qRT-PCR (Figure 5C). The expression profile of these genes closely mimicked the gene expression patterns from the microarray study, which validates both the public microarray data and our downstream bioinformatics analysis. Furthermore, as our qRT-PCR analysis contains many more data points compared to the previous study, the expression patterns of the genes are more thorough than the previous microarray results.

## DISCUSSION

We utilized a newly developed statistical and computational pipeline to examine the process of endocrine resistance in breast cancer and found novel underlying gene associations. First, we compared the data from patients resistant and sensitive to the endocrine therapies with publicly available gene expression data from cell-based models. We found LTED MCF7 cell model to be the closest to the patient tumor data based on full microarray chip gene expression. Next, we analyzed the time course expression in LTED cells during the process of acquiring endocrine resistance. Using our statistical pipeline, which is designed for identifying dynamically significant genes and clustering time-resolved expression [18, 19], we compared the DRGs to the gene expressions of patient tumor samples from endocrine-resistant and -sensitive tumors, finding a group of 318 genes as potential drivers of endocrine-resistance development. Using multiple bioinformatics approaches, we narrowed down the candidate genes list to 34 genes from four major modules, primarily from modules 1 and 2. Analyzing genes in the context of gene-gene networks enabled us to select the genes with a higher probability of being highly integral to resistance functions. These candidate genes are potential targets for developing potent therapies for endocrine-resistant breast cancer. The expression patterns of several candidate genes were further validated in biological settings by developing an LTED cell line.

Twenty out of the 34 genes were from the first module, which showed a slight decrease in expression by day 30, followed by significant upregulation afterward, which correlates to the growth rates of MCF7 cells under the development of endocrine resistance. As anticipated, the genes in module 1 were primarily associated with DNA replication and repair mechanisms, emphasizing the importance of these mechanisms in the replication and growth of cancer cells. Minichromosome maintenance complex genes *MCM2*, *3*, *4*, *6*, and *7* were identified in Module 1. Overexpression of *MCM2*, *MCM3*, *MCM4*, and *MCM6* is associated with luminal B, HER2^+^, and triple-negative breast cancers [39, 40]. *RAD51*, is known to be related to overall poor survival and endocrine resistance in breast cancer and is also linked to regulation of metastasis in TNBC [32, 41]. Among other genes in module 1 is replication factor C subunit 3 (RFC3), which is essential for the homologous DNA pairing and strand exchange. Downregulation of *RFC3* has been shown to attenuate cell proliferation, migration, and invasion in TNBC [42, 43]. The cyclin-dependent kinases (CDKs) are critical regulatory enzymes governing cell cycle transitions and play an essential role in the development of endocrine resistance in some breast cancer cases [10, 44]. While CDK 4/6 are the most studied members of the CDK family in relation to endocrine-resistant breast cancer, our analysis revealed another member of this family, *CDK1* to be important for both endocrine resistance and TNBC. Notably, CDK1 is required for the initiation of mitosis and cell proliferation, and its inhibition in TNBC led to a decrease in cell viability and an increase in cell apoptosis [45].

Among the remaining modules, 14 genes were identified, with 11 of them captured in module 2. The majority of these genes are already known to be related to breast cancer in some way, yet their connection to endocrine resistance and TNBC has not been discovered yet, calling for further experiments. Among the genes in module 2, the network analysis suggested *ELAVL1*, *GABARAPL2*, and *CAV1* as the main regulators of all other genes. Among the downregulated genes in module 2 was also protein phosphatase 1A (PPM1A). It was downregulated in both endocrine-resistant breast cancer and TNBC. This protein is a member of the protein phosphatase 2C family of Ser/Thr protein phosphatases and has been shown to regulate mitogen-activated protein kinase cellular signaling pathways as well as proliferation, cell invasion, and migration [46, 47]. Recently, Mazumdar et al. showed that this protein is significantly downregulated in ER-negative breast cancers and that its upregulation suppresses *in vitro* and *in vivo* growth of TNBC cells [48], which supports our findings.

Through identification of the candidate genes, we address a gap in knowledge regarding genetic factors underlying endocrine-resistant and triple negative breast cancers. As these two subtypes of breast cancer are the most fatal breast cancers with no known effective therapeutic approaches available to date, research on underlying genetic factors is of great importance. Here, we found a group of candidate genes that are significant dynamic genes and potential master regulators in the process of endocrine resistance. With further study, they may be potential targets for the treatment of endocrine-resistant breast cancers. Moreover, the majority of these genes are also significantly regulated in TNBC compared to luminal A breast cancer, suggesting that endocrine-resistant breast cancer and TNBC share mechanism similarity. Therefore, these genes may also be potential therapeutic targets for TNBC. Still, more than 1 200 genes were significantly regulated in TNBC and endocrine resistance development in LTED cells but not in endocrine-resistant breast cancer patients. This area of dissent may be due to the limitation that data from endocrine-resistant patients only show the comparisons between already resistant and sensitive tumors. Therefore, expression measurements may not represent genes important to establishment of endocrine resistance. Because we cannot determine whether the endocrine-resistant patients had already developed resistance by the time of the endocrine therapy exposure, we cannot verify the “stage” of resistance. In contrast, our time course approach analyzed the process of developing endocrine resistance, not just the endpoints.

Our novel statistical and computational analysis approach, combined with the use of multiple data sources, from cell modelling to patients, reveal new potential for developing effective therapeutic approaches toward various diseases. Further biological experiments are warranted to confirm a measurable degree of importance of these genes. While we utilized existing time course expression and patient tumor sample datasets, future datasets for both types will enhance the ability to validate and increase the reliability of our findings.

Our analysis identified novel candidate genes with potential significance in endocrine-resistant breast cancer as well as TNBC, which opens new doors for designing novel therapeutic approaches for endocrine-resistant breast cancer and TNBC. Potential future studies should focus on translational aspects on this research through deleting or overexpressing these genes in breast cancer models *in vivo* and explore the effects, as these genes can potentially lead to therapeutics for breast cancer.

## MATERIALS AND METHODS

### Data acquisition and processing

The microarray data of GSE20361, GSE87411, and GSE111151 were downloaded from GEO, the public functional genomics repository [15–17]. Data from GSE111151 and GSE87411 were analyzed with Limma. Using setting defaults, we identified the differentially expressed genes from both datasets for downstream analysis. To generate the heatmap, the log-fold-change values of the datasets and the “ComplexHeatmap” package in R were used. To identify the genes significantly regulated in TNBC compared to luminal A breast cancer, we used UALCAN [49]. Each gene was checked for its expression in the TNBC subtype compared to luminal A subtype. Using significance level of 0.05, those with significant differential expression were selected for further analysis.

Data from GSE20361 were analyzed using a MATLAB-based pipeline to identify DRGs, described as below. Individual GRMs were analyzed using the Metascape tool for gene enrichment analysis with default parameters [50]. To generate the gene-gene and protein-protein interaction networks, gene lists were analyzed using NetworkAnalyst, ToppGene, ToppFun, and Funcoup using the default parameters [23–26]. For tools that required a training gene set, we gathered known genes for endocrine-resistance development from literature.

### Dynamic gene expression analysis pipeline

As part of the analysis, we used a pipeline analysis method previously published [18, 19]. The pipeline analysis streamlines key processing performed in MATLAB. First, spline smoothing is applied to centered expression trajectories. Second, statistical hypothesis testing identifies a set of dynamic response genes (DRGs) that exhibit signiﬁcant change over time. Third, these DRGs are clustered into gene response modules (GRMs), subsets of DRGs with similar time course expression patterns.

One key assumption of the methodology is that a small fraction of the genes respond to external stimuli. Implicit in this assumption, we assume a large fraction of the genes maintain constant expression over time. We assume our observations of both responsive and unresponsive trajectories are distorted by noise. We present the centered expression profile of the *i^th^* gene, belonging to experimental condition *j*, as *X_i,j_*(*t*), a smooth function of time. Each measurement becomes a discrete realized value of the function, distorted by independent and identically normally distributed noise *E_i,j_*(*t_k_*) with mean 0 and variance σ^2^,


$$\begin{array}{c} Y_{i,j}(t_{k})-\mu_{i,j}=X_{i,j}(t_{k})+E_{i,j}(t_{k}),\\ \text{for }i=1,\;\ldots \;,\;n\;j=1,\;\ldots \;,\;N\;and\;k=1,\;\ldots \;,\;K_{i,j} \end{array}$$

where *n* is the number of genes, *N* is the number of subjects, and *K_i,j_* is the number of time points observed for each gene.

Spline smoothing is employed to obtain the functional entity *X_i,j_*(*t*). To obtain a smoothing regularity parameter, a subset of genes that exhibit large interquartile ranges, the top 200 most-responsive genes are used to minimize generalized cross-validation. After smoothing was applied, statistical hypothesis testing was conducted using F-tests


$$H_{0}\text{ ​}:\;X_{i,j}(t)=0\text{ vs }H_{a}\;:\;X_{i,j}(t)\neq 0$$

to identify DRGs. Any gene with significant results is classified as a DRG; however, due to computational demand, only the top 3000 DRGs were selected for further analysis, as justified by simulation study [18].

In the next step, the DRGs that exhibit similar expression patterns over time are clustered into temporal gene response modules (GRMs) using iterative hierarchal clustering (IHC) with Spearman correlation threshold of 0.7. The IHC algorithm can identify inhomogeneous clusters, capturing trends at many scales by selecting optimal cluster size, resulting in large and also very small clusters. From a biological perspective, this step is interpreted as genes tend to act in collaboration. From a computational perspective, the step reduces dimension of the problem. Additional methodology description is found in previous works [18, 19].

### LTED cell line development

MCF7 cells were cultured in estrogen-depleted growth media (phenol red-free RPMI1690 medium (Thermo Fisher Scientific, Waltham, MA) supplemented with 5% charcoal-stripped serum (CSS) and 10 μg/ml of Insulin (Thermo Fisher Scientific) for extended periods. Cell samples were collected every 7 days and used for downstream analysis.

### Proliferation assay

Both parental and LTED MCF7 cells were cultured in a 96-well plate. After 24 hours, the cells were treated in triplicates with E_2_ in RPMI1690 plus 5% CSS. The treatment was repeated every other day and at the end of the seventh day, then the plates were frozen for the assay. FluoReporter blue fluorometric dsDNA quantification kit (Invitrogen, Waltham, MA, USA) was used per the manufacturer’s protocol. The excitation/emission wavelength of 360/460 nm were read and normalized to the control wells.

### qRT-PCR analysis

mRNA abundance was evaluated by qRT-PCR as described [19]. Relative mRNA levels were calculated by using the comparative CT method normalized to cyclophilin. The primers were designed using Primer Express Software (Applied Biosystems, Waltham, MA, USA) as shown in Supplementary Table 2.

## SUPPLEMENTARY MATERIALS



## Abbreviations

BC: breast cancer
CDK: cyclin-dependent kinase
DRG: dynamic response gene
GEO: gene expression omnibus database
GRM: gene response module
GSE: gene series record in GEO database
IHC: iterative hierarchical clustering
LTED: long-term estrogen deprivation
qRT-PCR: quantitative reverse transcription polymerase chain reaction
TNBC: triple negative breast cancer
