# Supplementary material for: A novel group of genes that cause endocrine resistance in breast cancer identified by dynamic gene expression analysis
Source: Oncotarget. 2022 Apr 6;13:600–13. doi: 10.18632/oncotarget.28225 (PMC8986262; doi:10.18632/oncotarget.28225)
Supplement: Supplementary file 1 [file oncotarget-13-28225-s001.pdf]

A novel group of genes that cause endocrine resistance in breast cancer identified by dynamic gene expression analysis

SUPPLEMENTARY MATERIALS

Supplementary Table 1: List of candidate genes underlying endocrine resistance development and progression

Module	Gene symbol	Gene ontology
1	CDK1	Cell cycle
1	FEN1	DNA repair
1	H2AFX	DNA repair
1	MCM2	Cell cycle
1	MCM3	Cell cycle
1	MCM4	Cell cycle
1	MCM6	Cell cycle
1	MCM7	Cell cycle
1	MSH2	DNA repair
1	MSH6	DNA repair
1	POLD1	DNA repair
1	PRKDC	DNA repair and Immune response
1	RAD51	DNA repair
1	RECQL4	DNA repair
1	RFC3	DNA repair
1	RFC5	DNA repair
1	SMC4	Cell cycle
1	TCF3	DNA binding
1	TMPO	Cell-cell adhesion
1	TUBB	Cell cycle
2	CAV2	ER organization and cell differentiation
2	CCNT2	Cell cycle
2	CD59	ER to Golgi vesicle mediated transport
2	ELAVL1	Regulation of RNA stability
2	GABARAPL2	Autophagy
2	MYOF	Plasma membrane repair
2	PPM1A	Cell cycle
2	PTPN3	Cell cycle
2	SH3GLB2	Cell-cell adhesion
2	TBC1D20	ER to Golgi vesicle mediated transport
2	USP47	Cell cycle
3	ATG3	Autophagy
3	CCNE1	Cell cycle
4	MFAP4	Cell-cell adhesion

Supplementary Table 2: List of the primers used in qPCR

Gene	Forward primer	Reverse primer
**CDK1**	GGAAACCAGGAAGCCTAGCATC	GGATGATTCAGTGCCATTTTGCC
**MCM2**	TGCCAGCATTGCTCCTTCCATC	AAACTGCGACTTCGCTGTGCCA
**CAV1**	CCAAGGAGATCGACCTGGTCAA	GCCGTCAAAACTGTGTGTCCCT
